# Machine Learning, Deep Learning, and Data Preprocessing Techniques for Detecting, Predicting, and Monitoring Stress and Stress-Related Mental Disorders: Scoping Review

**DOI:** 10.2196/53714

**Published:** 2024-08-21

**Authors:** Moein Razavi, Samira Ziyadidegan, Ahmadreza Mahmoudzadeh, Saber Kazeminasab, Elaheh Baharlouei, Vahid Janfaza, Reza Jahromi, Farzan Sasangohar

**Affiliations:** 1 Department of Industrial and Systems Engineering Texas A&M University College Station, TX United States; 2 Department of Computer Science and Engineering Texas A&M University College Station, TX United States; 3 Zachry Department of Civil and Environmental Engineering Texas A&M University College Station, TX United States; 4 Harvard Medical School Harvard University Boston, MA United States; 5 Department of Computer Science University of Houston Houston, TX United States

**Keywords:** machine learning, deep learning, data preprocessing, stress detection, stress prediction, stress monitoring, mental disorders

## Abstract

**Background:**

Mental stress and its consequent mental health disorders (MDs) constitute a significant public health issue. With the advent of machine learning (ML), there is potential to harness computational techniques for better understanding and addressing mental stress and MDs. This comprehensive review seeks to elucidate the current ML methodologies used in this domain to pave the way for enhanced detection, prediction, and analysis of mental stress and its subsequent MDs.

**Objective:**

This review aims to investigate the scope of ML methodologies used in the detection, prediction, and analysis of mental stress and its consequent MDs.

**Methods:**

Using a rigorous scoping review process with PRISMA-ScR (Preferred Reporting Items for Systematic Reviews and Meta-Analyses extension for Scoping Reviews) guidelines, this investigation delves into the latest ML algorithms, preprocessing techniques, and data types used in the context of stress and stress-related MDs.

**Results:**

A total of 98 peer-reviewed publications were examined for this review. The findings highlight that support vector machine, neural network, and random forest models consistently exhibited superior accuracy and robustness among all ML algorithms examined. Physiological parameters such as heart rate measurements and skin response are prevalently used as stress predictors due to their rich explanatory information concerning stress and stress-related MDs, as well as the relative ease of data acquisition. The application of dimensionality reduction techniques, including mappings, feature selection, filtering, and noise reduction, is frequently observed as a crucial step preceding the training of ML algorithms.

**Conclusions:**

The synthesis of this review identified significant research gaps and outlines future directions for the field. These encompass areas such as model interpretability, model personalization, the incorporation of naturalistic settings, and real-time processing capabilities for the detection and prediction of stress and stress-related MDs.

## Introduction

### Background

Mental health has become a public health concern. According to the Institute of Health Metrics and Evaluation, in 2019, about 53 million people in the United States and about 1 in 8 individuals worldwide (about 1 billion people) have at least 1 mental health disorder (MD) [1]. An MD is defined as an impairment in a person’s cognition, emotional control, or behavior patterns that has clinical significance and is often linked to distress or functional impairment [2]. MDs severely limit people’s daily functioning and can be fatal [3,4]. In 2019, mental health (MH) problems accounted for 6.6% of all disability-adjusted life years in the United States, making it the fifth most significant cause of disability overall [1,5].

Some of the more prevalent MDs are anxiety disorders, depression or mood disorders, bipolar disorders, psychotic disorders (including schizophrenia), eating disorders, social disorders, disruptive behavior, and addictive behaviors [2]. In 2019, anxiety and depression have been the most prevalent forms of MDs (301 and 280 million people affected worldwide, respectively). Anxiety disorder encompasses emotions of concern, anxiety, excessive fear, or associated behavioral problems that are severe enough to affect everyday activities [2]. Symptoms include an unproportionate level of stress compared to the significance of the triggering event, difficulty in putting worries out of one’s mind, and nervousness [6,7]. Generalized anxiety disorder, panic attacks, social anxiety disorder, and posttraumatic stress disorder (PTSD) are all examples of different types of anxiety disorders [2,8]. Depression is characterized by a long-lasting sadness and a lack of desire to be active. One of the main symptoms of depression is the inability to enjoy or find pleasure in most of one’s daily activities as well as feeling sadness, anger, or emptiness [2,9]. A depressive episode typically lasts for at least 2 weeks. In addition, a loss of self-worth, feelings of hopelessness for the future, and suicidal thoughts are indicators and symptoms of depression. People who are depressed are more prone to commit suicide [2,9,10].

Stress is categorized into distress, which typically has chronic negative effects on health, and eustress, which is short-term and positively influences motivation and development [11]. Throughout this paper, the term stress is specifically used to denote distress rather than eustress. Mental stress has been shown to significantly contribute to developing and worsening anxiety and depression disorders [12-14]. Mental stress is the body’s natural response to various events in which a person feels that the demands of their external environment exceed their psychological and physiological resources for dealing with those demands [15]. Mental stress leads to an asynchrony between the sympathetic and parasympathetic nervous systems (SNS and PNS), which are the main divisions of the autonomic nervous system [16] and serve an important role in regulating vital biological activities [17,18]. The SNS is an integrative system that responds to potentially dangerous circumstances. Activation of the SNS is part of the system responsible for controlling “fight-or-flight” responses. The PNS is responsible for the body’s “rest-and-digest” processes.

Given the important role and impact of stress in MDs, previous research has investigated various qualitative and quantitative methods to measure and monitor stress to inform effective stress mitigation approaches. While majority of stress literature relies on self-reported measures, recent literature has used physiological variables such as heart rate (HR); HR variability (HRV) [19-23]; and behavioral data (eg, speech, movement, and facial expressions) [24] to understand changes to SNS and PNS associated with stress. The recent advances in sensor and mobile health technologies have resulted in the emergence of big data related to MH, as well as advanced bioinformatics methods, tools, or techniques to use such data for modeling or inference. One such tool that has recently emerged as a robust, rapid, objective, reliable, and cost-efficient technique for studying chronic illnesses and MDs is machine learning (ML). ML uses advanced statistical and probabilistic techniques to construct systems that can automatically learn from data. Several characteristics of ML make it suitable for applications in MH monitoring including significant pattern recognition and forecasting capabilities [25], the capacity to extract crucial information from various data resources and the opportunity to create personalized experiences [25], and the ability to analyze large amounts of data in a short time [26]. As such, ML has gained popularity and has been applied to MH data to enable detection, monitoring, and treatment [27]. The objective of this research is to review the literature to summarize and synthesize the application of ML in the detection, monitoring, or prediction of stress and stress-related MDs, in particular, anxiety and depression. This paper documents method-specific findings such as data types, preprocessing methods, and different algorithms used, as well as the type and characteristics of studies that used ML.

Traditional statistical methods, such as linear regression, logistic regression (LR), 1- or 2-tailed *t* tests, and ANOVA [28], have been widely used in the past to detect and analyze stress and stress-related MDs. These methods have proven useful in specific contexts, such as comparing means of different groups or modeling linear relationships between variables. As demonstrated by Machado et al [21], Adjei et al [22], Yoo et al [23], Chen et al [24], and Jordan and Mitchell [25], these methods have provided valuable insights in situations wherein the data are relatively simple and adhere to the underlying assumptions of the statistical techniques. However, when faced with complex, high-dimensional MH data, which have become increasingly available, thanks to advancements in technology and data collection techniques, these traditional statistical methods might not be sufficient. The limitations of these methods stem from their inherent simplicity and the assumptions they rely on, which might not hold true in the context of MH data. For example, linear regression and LR analyses assume linear relationships between variables, while *t* tests and ANOVA require specific assumptions about the data distribution. These assumptions may not be applicable in the case of intricate and heterogeneous MH data, potentially leading to inaccurate or incomplete conclusions.

Advanced data analytics methods, such as ML, offer a more powerful and flexible alternative to traditional statistical methods. ML algorithms, with their significant pattern recognition and forecasting capabilities [25], are capable of capturing complex, nonlinear relationships between variables and can adapt to various data distributions. These capabilities enable ML techniques to provide more accurate and insightful predictions, classifications, and associations in the context of MH data [29]. In addition, ML algorithms can handle large-scale, high-dimensional data more efficiently than traditional methods, allowing researchers to analyze vast amounts of information from diverse sources, such as eHealth records, wearable devices, and web-based platforms [26]. This capacity for handling big data is crucial for understanding the multifaceted nature of MDs and developing tailored interventions. ML techniques also offer the advantage of automation and adaptability, allowing them to continuously learn and improve as new data become available [25]. This iterative learning process enables the development of more sophisticated and accurate models for detecting, monitoring, and predicting stress and stress-related MDs over time.

While traditional statistical methods have contributed significantly to our understanding of stress and stress-related MDs in specific contexts, the growing complexity and volume of MH data necessitate the adoption of advanced data analytics methods such as ML. By leveraging the power of ML, researchers can gain deeper insights into the underlying patterns and relationships between stress and MDs [29], ultimately leading to the development of more effective stress mitigation approaches and improved care for individuals who have anxiety, depression, and other MDs.

Acknowledging the substantial contributions of traditional statistical methods, it becomes evident that the escalating complexity and scale of MH data demand the adoption of more sophisticated approaches such as ML. This advancement stands not as a replacement but as an essential evolution in the analytical toolbox available to researchers. As this paper delves into the myriad ways that ML has been applied to MH, particularly in the realms of stress, anxiety, and depression, it seeks to consolidate the current knowledge on the subject.

### Objectives

By examining the types of data, preprocessing methods, and the algorithms used in existing studies, this review aspires to offer a detailed synthesis of the field. It aims to provide a clearer understanding of ML’s effectiveness in the detection, monitoring, and prediction of MDs, setting a foundation for future research and the enhancement of therapeutic strategies for those impacted by these conditions.

## Methods

### Protocol and Registration

This scoping review adhered to the PRISMA-ScR (Preferred Reporting Items for Systematic Reviews and Meta-Analyses extension for Scoping Reviews) guidelines [30]. No formal review protocol was registered due to the exploratory nature of this study, which aimed to map out existing research rather than address a prespecified hypothesis. This approach aligns with the methodological flexibility often required in emergent areas of research.

### Eligibility Criteria

We included studies published in English from 2017 to 2022 that used ML techniques to evaluate MDs, specifically focusing on stress and stress-related conditions. Studies were excluded if they did not use ML as the primary analysis method or if they were published in languages other than English.

### Information Sources

The literature search involved databases such as EI Engineering Village, Web of Science, ACM Digital Library, and IEEE Xplore. Additional sources were identified through contact with experts and review of references in relevant articles.

### Search Strategy

A comprehensive search was conducted using a combination of keywords related to ML and MDs (Textbox 1). The search strategy was designed to capture a broad spectrum of ML applications within this field. The full search list from all databases is available in Multimedia Appendix 1 [31-50].

**Keywords and search strategy for articles since 2017 (last 5 years).**

**Search strategy**

First keyword: predict OR detect

AND

Second keyword: mental health OR mental disorder OR depression OR anxiety OR stress

AND

Third keyword: machine learning OR deep learning OR data mining OR pattern classification OR artificial intelligence OR neural networks

### Study Selection, Inclusion, and Exclusion Criteria

**Figure 1:**
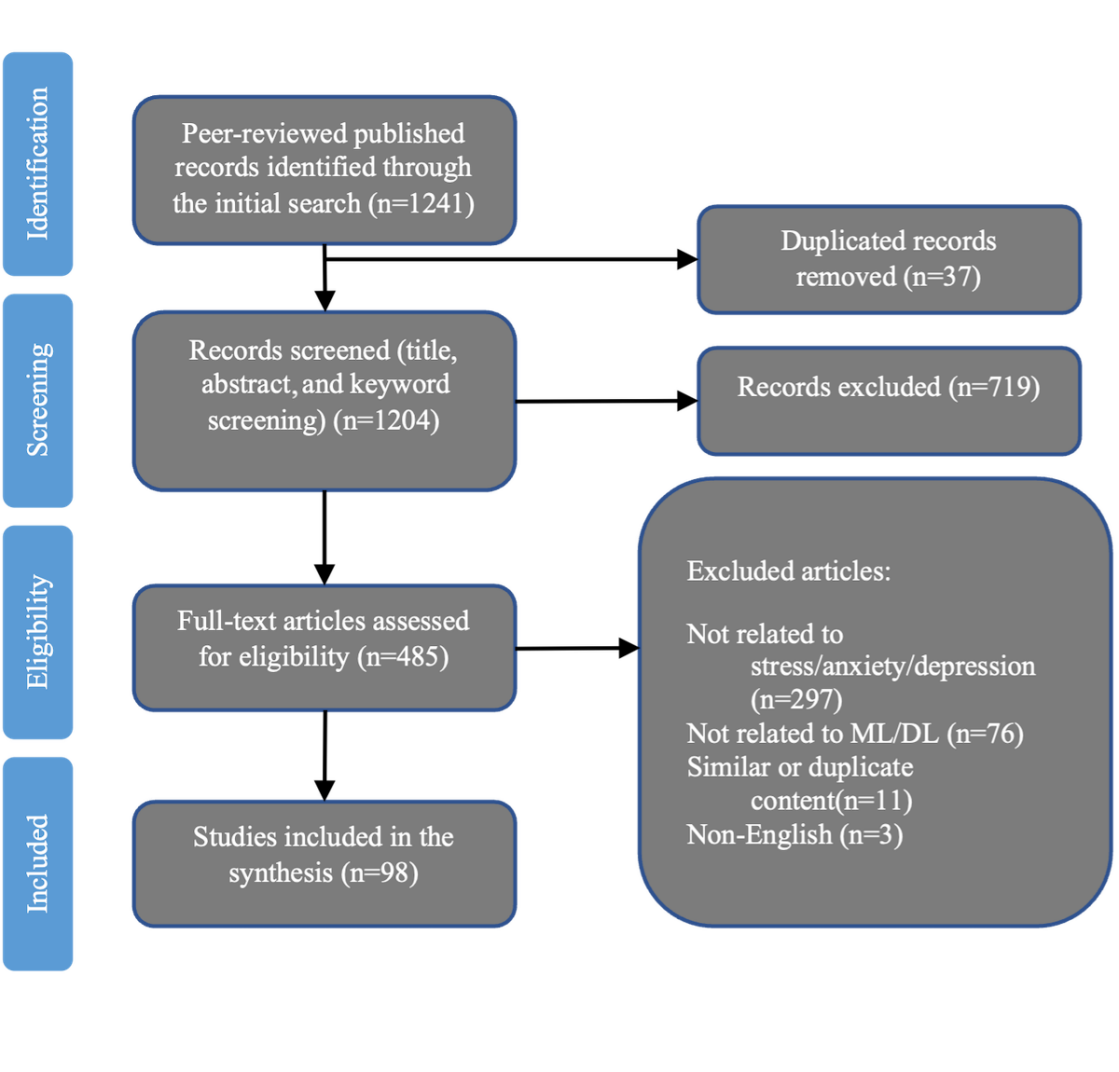
Preferred items for scoping literature review and meta-analysis flowchart (modified from Tricco et al [30]). DL: deep learning. ML: machine learning.

Articles that did not fully use ML for stress or stress-related MDs evaluations were excluded from the research. Studies published in languages other than English were also excluded. The initial search yielded 1241 results. After duplicate articles were deleted and eligibility was confirmed using Rayyan (Qatar Computing Research Institute) [51], 1204 (97.02%) articles remained. After applying the exclusion criteria, 98 (8.14%) papers were selected for full review (Figure 1).

### Data Charting Process

Data charting was conducted by 2 reviewers independently using a standardized form, which had been pretested on a subset of included studies. Discrepancies were resolved through discussion or consultation with a third reviewer. Study authors were contacted for clarification or additional data where necessary.

### Data Items

Data extracted included publication year, study design, population characteristics, ML techniques used, outcomes measured, and key findings. Other variables sought included data preprocessing methods and performance metrics of the ML models. Simplifying assumptions, such as considering different ML algorithms within the same family as a single technique, were made to facilitate synthesis.

### Synthesis of Results

Data were synthesized descriptively, and the findings were grouped by ML techniques, data type, and preprocessing techniques. Where possible, quantitative performance metrics were extracted or derived. Results were analyzed in the context of the overall study designs and populations to highlight trends and identify gaps in the current research landscape. No formal critical appraisal or quantitative meta-analysis was conducted due to the diversity of the included studies and the scoping nature of this review.

## Results

In this section, types of data, preprosessing techniques, and ML techniques used on the data in the literature have been reviewed and compared with the existing literature.

### Types of Data

#### Overview

Various data types were used in the studies (n=98) that used ML algorithms for stress and stress-related MDs. Studies used questionnaires (n=31, 32%); HRV (n=25, 26%); skin response (eg, skin temperature, skin conductance, etc; n=24, 24%); photoplethysmogram (PPG; n=21, 21%); electrocardiogram (ECG; n=19, 19%); HR (n=17, 17%); electroencephalogram (EEG; n=9, 9%); acceleration or body movement (n=8, 8%); text data (n=7, 7%); respiratory signals (n=7, 7%); electromyogram (EMG; n=3, 3%); eye tracking (n=3, 3%); speech signals (n=3, 3%); and others (n=4, 4%) including audio signals (n=2, 2%), blood pressure (BP; n=1, 1%), and hormones (n=1, 1%). Table 1 shows the distribution of the type of data used for stress detection using ML techniques.

**Table 1:** Number of articles by type of data (n=98).

Type of data	Articles, n (%)
Questionnaire	31 (32)
HRV^a^	25 (25)
Skin response	24 (24)
HR^b^	17 (17)
EEG^c^	9 (9)
Body movement	8 (8)
Respiratory	7 (7)
Text	7 (7)
EMG^d^	3 (3)
Eye-tracking	3 (3)
Speech signals	3 (3)

^a^HRV: heart rate variability.

^b^HR: heart rate.

^c^EEG: electroencephalogram.

^d^EMG: electromyogram.

#### Heart Measures

Heart metrics are primarily used for stress detection and are typically gathered through 2 main methods: ECG and PPG. ECG is a noninvasive diagnostic test that records the heart’s electrical activity, while PPG is a noninvasive optical technique that detects changes in blood volume within the tissue’s microvascular bed. By using these methods, it is possible to measure various heart-related parameters, including HR, as well as time and frequency domain features of HRV and BP.

#### HRV Measures

**Figure 2:**
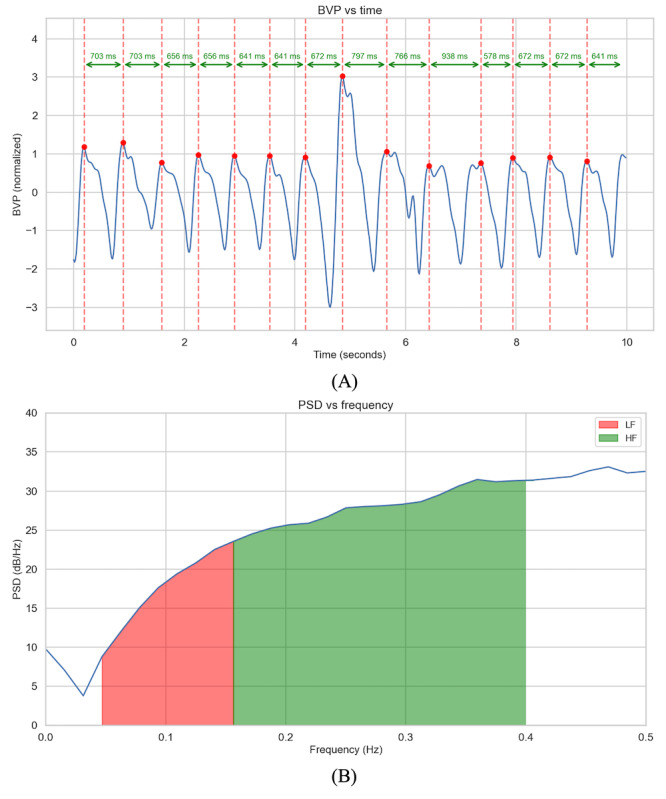
Depiction of heart’s beat-to-beat measurements using blood volume pulse (BVP) signal (A) and power spectral density (PSD) of RR intervals (the signal is bandpass filtered with cutoff frequencies of 0.04 Hz and 0.4 Hz) (B). HF: high frequency; LF: low frequency.

HRV (n=25, 26%) has been used to assess MH issues, such as stress, anxiety, and depression, due to its rich time and frequency domain features [53]. The blood volume pulse signal is another effective method for capturing HRV features, as it represents the heart’s beat-to-beat volume changes. From the blood volume pulse signal, time-domain measures such as the root mean square of successive RR interval differences (RMSSD), SD of neural network (NN) intervals, and SD of RR intervals can be derived. In addition, the frequency domain aspects of HRV, including total power (frequencies <0.4 Hz), low frequencies (LFs; ranging from 0.04 to 0.15 Hz), and high frequencies (HFs; between 0.15 and 0.4 Hz), reflect the autonomic nervous system’s dynamics during beat-to-beat measurements of the HR (Figure 2) [54,55]. These HRV measures, both in the time and frequency domains, provide a nuanced view of the physiological underpinnings associated with various MH conditions.

#### HR Measures

One of the most important indicators of stress is an abrupt increase in HR (n=17, 17%). Among the physiological signals, HR is among the top measures that explain stress in ML models, and it has been used in different studies with almost all ML algorithms [56-58].

#### BP Measures

BP (n=1, 1%) can be obtained by pulse transit time or by pressure cuffs [59]. Stressful conditions create an influx of hormones that increase HR and constrict blood vessels, leading to a temporary BP elevation [60]. In most cases, BP recovers to its prestress level after the stress response diminishes [61]. Schultebraucks et al [62] used systolic BP as one of the measures in predicting one’s level of susceptibility to PTSD.

#### EEG Data

EEG (n=9, 9%) detects brain electrical activity. Compared with other brain mapping techniques for stress detection, it is more practical due to several factors including affordability, noninvasiveness, nonintrusiveness, and, most importantly, its high temporal resolution [63]. The high temporal resolution of EEG makes it appropriate for real-time stress detection, as well as deep learning (DL) approaches, which require large data sets for training [63-67].

The most commonly used EEG features for the detection of stress are the power of different frequency bands (α, 8-13 Hz; β, 12.5-30.0 Hz; θ, 4.5-7.5 Hz; γ, 30-40 Hz), average and SD of a specific time window of EEG signal, and time-frequency features obtained by discrete wavelet transform algorithm [67-69]. It has also been shown that statistical features of EEG signals, such as kurtosis and entropy, are useful features in stress prediction using ML algorithms [66]. Moreover, power spectral density (PSD), correlation, divisional asymmetry, rational asymmetry, and power spectrum are other EEG features that have been used in different studies for stress detection [70].

Since EEG signals are collected from the scalp, they include excessive noise and have high uncertainty. Therefore, signal processing and feature selection or extraction are important steps while dealing with EEG data. Several well-developed methods are available for treating the EEG data. Among them, latent space derived from auto-encoders and signal reconstruction techniques such as artifact subspace reconstruction (ASR) are well-known methods that can be applied on EEG data to significantly reduce the artifacts [65]. These methods are also fast enough to make real-time detection feasible.

The amygdala and hippocampus are the parts of the brain that have the major responsibility for human reactions to stress [71]. Brain activity caused by stress in those regions would affect the prefrontal cortex. Studies collecting data from the prefrontal cortex have also verified that EEG data from this brain region can be used for stress detection [72]. EEG can be collected from the prefrontal cortex using off-the-shelf EEG recording products such as Muse and Neurosky Mindwave [66,69,70,72].

#### Eye Tracking

Eye-tracking features (n=3, 3%) can be indicators of stress. For example, to diagnose the level of stress, the changes in the striations of muscle material in the iris as a response to stress can be used as features for ML algorithms. In other words, pupil diameter, which is controlled by iris sphincter muscles, can be used as a feature [73]. Other eye-tracking features that have been used for stress detection are visual fixations, saccade movements, pupil size, microsaccades, and the number of eye blinks in a specific time window during a certain task [74-76].

#### Skin Response

A skin response (n=24, 24%) can be defined as a stimulus-regulated electrodermal response and is typically measured using electrodes placed on the fingertips or hands. Skin response is usually associated with an increase in sympathetic activity on inducing stress events [77]. The skin becomes a better conductor of electricity when it is stimulated either externally or internally by physiologically stimulating factors, including stressful conditions [78].

#### Respiratory Signals

Mental stress can affect different respiratory cycle phases and breathing patterns (n=7, 7%) [79,80]. For example, it has been discovered that stress had no impact on overall breath duration (respiration rate) but that exhalation periods were longer and pause periods were shorter in the stress experiment than in the neutral condition [81].

On the basis of the findings of several studies, it can be concluded that respiratory signal is one of the top contributing factors in the explanation of stress in ML models. The most common time-domain respiratory signal features that are extracted for stress detection are root mean square, IQR, and mean square differences between adjacent elements of breathing rate and blood oxygenation levels. The most commonly used frequency domain features of the respiratory signal are the power of LFs (<2 Hz), the power of HFs (>2 Hz), and the ratio of the power of LFs over the power of HFs (LF and HF) [58,62,63,82-84].

#### EMG Data

EMG (n=3, 3%) detects the electrical activity of muscles at rest, during a modest contraction, and during a strong contraction [85]. Similar to acceleration data, several studies have shown that using EMG data can help increase the performance of ML models trained on ECG data. The action potential intrigued in the EMG during stress can reduce the variance for decision-making of classification models that use ECG [58,86,87].

#### Hormones

It has been shown that stress can alter the levels of glucocorticoids, catecholamines, growth hormones, and prolactin in the bloodstream. Therefore, in ML models, levels of hormones such as cortisol, dehydroepiandrosterone sulfate, thyroid-stimulating hormone, free triiodothyronine, and free thyroxine can be used as predictors for the detection of stress-related disorders (n=1, 1%) [62].

#### Acceleration and Body Movement

Mental stress may cause a wide variety of behavioral and body movement symptoms such as shaking hands and feet, which can be measured by the acceleration data (n=8, 8%) [88]. Moreover, research has shown that people with a greater stress score had less variance in their activity level and body movements [89-91]. For example, in older adults, stressful life events can be related to a reduced rate of regular physical exercise [92]. Time and frequency features such as mean absolute deviation from the mean, the total power of acceleration, SD, the mean norm of acceleration, absolute integral, and peak frequency of each axis are the features of hand and body acceleration used for stress detection [31,57,93]. One practical characteristic of motion and acceleration data is that they can be used to identify sources of noise in other signals. For example, motion data can help distinguish stress from physical activity (eg, exercise) when other physiological measures, such as ECG, have uncertainty in prediction [94,95].

#### Audio and Speech Signals

#### Speech Signals

Using speech signals (n=3, 3%), it is feasible to diagnose and assess neurological disorders and MDs [96]. Moreover, studies have shown that like body acceleration and EMG, features of speech signals can make stress predictions of heart measurements more robust. The best explanatory parameters of speech signals are frequency domain parameters (eg, PSD, strongest frequency from fast Fourier transform) and time-frequency features such as Mel-frequency cepstral coefficient [56,97,98]. Since time-frequency measures are 2-dimensional measurements with a high number of samples, they make this signal suitable for use in convolutional NN (CNN) models of stress and depression detection [99].

#### Audio Signals

For laboratory-based studies, audio signals (eg, beeping sounds; n=2, 2%) can be used to stimulate stress events in participants [100,101].

#### Text Data

Social media content (n=7, 7%) is frequently subjected to reviews, opinions, and influence, as well as sentiment analysis. Natural language processing methods may be used to evaluate social networking posts and comments for mood and emotion to detect whether a user is stressed [102-108].

#### Questionnaire

Different questionnaires (n=31, 32%) are used for the diagnosis of stress and MDs including anxiety and depression. The scores from different items on these questionnaires can be used as dependent and independent variables in ML studies. The questionnaires mentioned here were selected based on their prevalence in the literature as well as their relevance to the ML outcomes being predicted. For instance, some studies have successfully leveraged scores from multiple questionnaires, such as the Diagnostic and Statistical Manual of Mental Disorders, Depression Anxiety and Stress Scale, Edinburgh Perinatal/Postnatal Depression Scale, Center for Epidemiological Studies-Depression survey, Mean Opinion Score, Hamilton Depression Rating Scale, State-Trait Anxiety Inventory, Posttraumatic Stress Disorder Checklist for Diagnostic and Statistical Manual of Mental Disorders, Beck Depression Inventory, Beck Anxiety Inventory*,* Hospital Anxiety and Depression Scale, Goldberg’s Depression Scale, self-reports, and clinician reports [109-126].

### Preprocessing Techniques

In this section, important preprocessing techniques that have yielded significant findings and how they are used to help the detection of stress and its related MDs have been reviewed.

#### Synthetic Minority Oversampling Technique

In the detection of stress and its related MDs, the number of samples for the stress or MD class is usually significantly lower than the nonstress or non-MD class. This imbalance in the number of samples for each class leads to a bias in prediction (toward the majority class). To correct for data bias, it is possible to oversample the underrepresented group. In stress detection studies using ML models, the synthetic minority oversampling technique is one of the most common approaches to boost the minority class, which creates new samples by synthesizing those already available in the data (by combining their features; n=3, 3%) [31,110,127].

#### Early Modality Fusion

**Figure 3:**
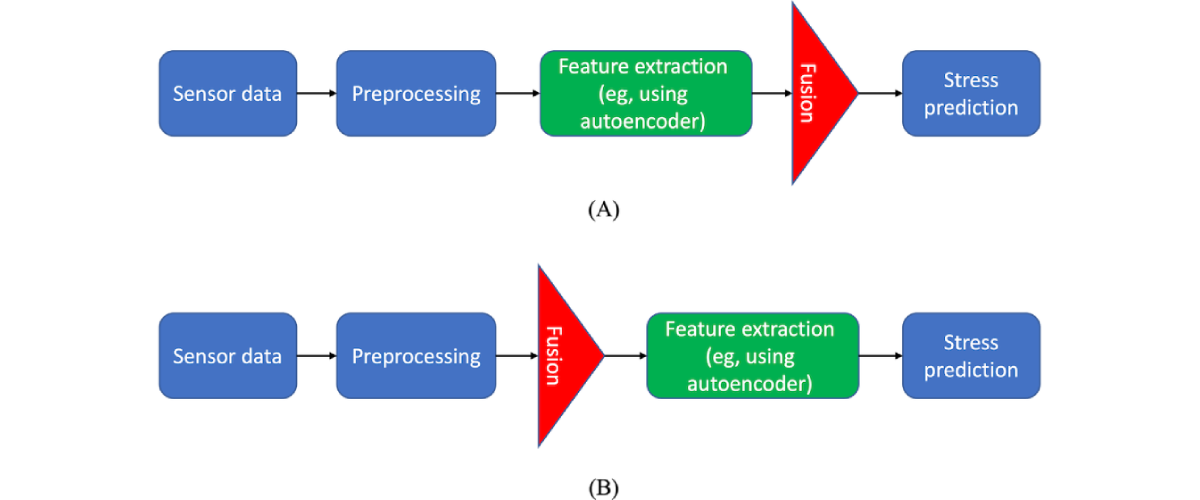
Early modality fusion (A) and late modality fusion (B).

In ML models used for the prediction of stress with a multimodal approach, it has been shown that early fusion of multimodal data before feature extraction is more effective and achieves a better performance (n=1, 1%). This is because early modality fusion better catches the important characteristics that are coherent with each other. For example, a study showed that combining different measures including skin response, skin temperature, and body acceleration before feature extraction outperforms the approach that extracts the features for each measure separately and combines them afterward (Figure 3) [128].

#### PSD Method

In physiological signals for stress detection, usually the power of the signal changes during the moments of stress. PSD (n=13, 13%) explains the frequency-based power distribution of a time series and reveals the locations of strong and weak frequency variation. Welch’s method is one of the most common approaches for calculating PSD [65]. PSD is often used in studies that include frequency domain HRV features for stress detection such as total HF or LF power [82-84,101,129-136].

#### ILIOU Method

In the detection of MDs, such as depression and anxiety, using ML techniques, having the least error rate is significantly important so that the person can take further actions appropriately. In this matter, the data preprocessing step has an important role in minimizing the noise and bias toward the false prediction. Iliou et al [114] proposed ILIOU (n=1, 1%), a data mapping and transformation method that identifies useful information for the detection of MDs, especially for depression. This method outperforms common data preprocessing techniques such as principal component analysis (PCA), evolutionary search algorithm, and isomap for the detection of depression.

#### PCA Method

PCA (n=3, 3%) is a method for lowering the dimensionality of such data sets while maximizing interpretability and minimizing loss of information. It does this by generating new variables that are uncorrelated and progressively optimize variance [58,90,123].

#### Independent Component Analysis

Independent component analysis (ICA; n=4, 4%) is a computational and statistical method for uncovering hidden elements underlying random variables, observations, or signals. This method is mostly used for removing artifacts from stationary signal noises of the multichannel data. ICA optimizes higher-order statistics such as kurtosis, while PCA optimizes the covariance matrix of the data, which reflects second-order statistics. In stress detection using physiological signals that contain stationary noises (eg, eyeblink noise in EEG) it is recommended to remove noises using ICA [63-65,67].

#### ASR Approach

ASR (n=1, 1%) is an adaptive approach for removing artifacts from signal recordings on the web or offline, mostly nonstationary signal noises. To identify artifacts based on their statistical qualities in the component subspace, a PCA on covariance matrices is repeatedly computed [137]. Since there are usually substantial nonstationary noises in the EEG data, in order to classify stress at multiple levels using EEG data, using ASR before classification is highly recommended [65].

#### Latent Growth Mixture Modeling

Growth mixture modeling (n=1, 1%) is to discover numerous hidden subpopulations, describe longitudinal development within each hidden subpopulation, and investigate variation in hidden subpopulations’ rates of change. Latent growth mixture models are gaining popularity as a statistical tool for estimating individual development over time and for probing the presence of latent trajectories, in which people belong to the trajectories that are not directly observable [62,138,139].

#### Dynamic Time Warping

**Figure 4:**
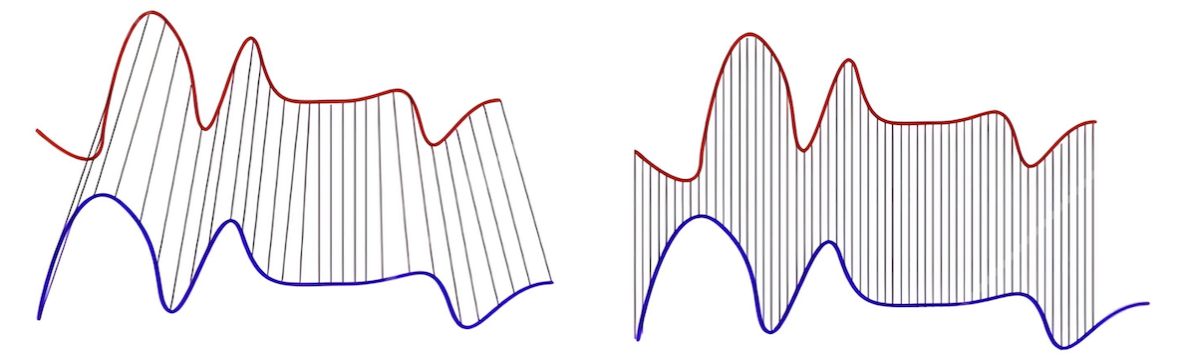
Dynamic time warping (left) versus Euclidian matching (right; modified from Portilla and Heintz [142]).

It is common practice to transform data from 2 time series into vectors and then compute the Euclidean distance between the resulting points in vector space to determine the degree of similarity or dissimilarity between the series, regardless of whether they vary in time or velocity. Dynamic time warping (DTW) method (n=1, 1%) can be applied to find such similarities that may exist between people in terms of their mood series. As an example, one may compare time series to find whether they match for stress, depression, or anxiety. Moreover, it can be used to forecast the mental condition of persons with substantially comparable series patterns [130,140]. The difference between DTW and Euclidian matching is that unlike Euclidean matching, DTW considers the distance of each point in one sequence to every point in the other sequence to determine the similarity between them (Figure 4).

#### Kalman Filter

The Kalman filter (n=2, 2%) is a technique for making predictions about unknown variables (eg, missing data) based on observable data. Kalman filters include 2 iterative steps, predict and update, that are used to estimate states using linear dynamical systems in state-space format. Iterative cycles of predict and update are performed until convergence is achieved [143]. Kalman filter has been used to handle the missing data for stress detection in some studies [144,145].

#### Autoencoders

Autoencoders (n=3, 3%) are a type of NNs that learn a representation of the data in lower dimensions than the original data (encoding) by regenerating the input from the encodings (decoding). For data with very high dimensionality, usually clustering is not optimized because of the noise present in the original data. Hence, it is an appropriate practice to use the encoded representation of the data, obtained by autoencoders, to have lower and more optimized dimensions for clustering [65,108,146].

#### Self-Organizing Map

**Figure 5:**
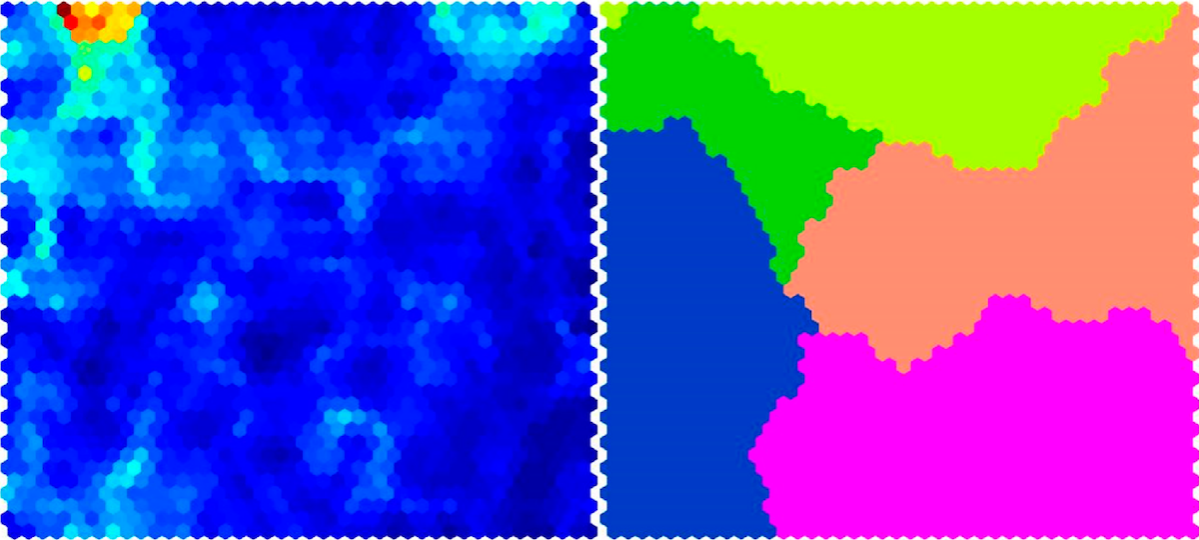
Representation of self-organizing map (SOM) before (left) and after mapping (right; modified from Cho et al [133]).

In ML, a self-organizing map (n=3, 3%) produces a low-dimensional, typically 2-dimensional, representation of a high-dimensional data set while preserving its topology by creating clusters. Therefore, it is possible to visualize and analyze high-dimensional data more easily (Figure 5) [107,133,147].

#### Wrapper Feature Selection Methods

**Figure 6:**
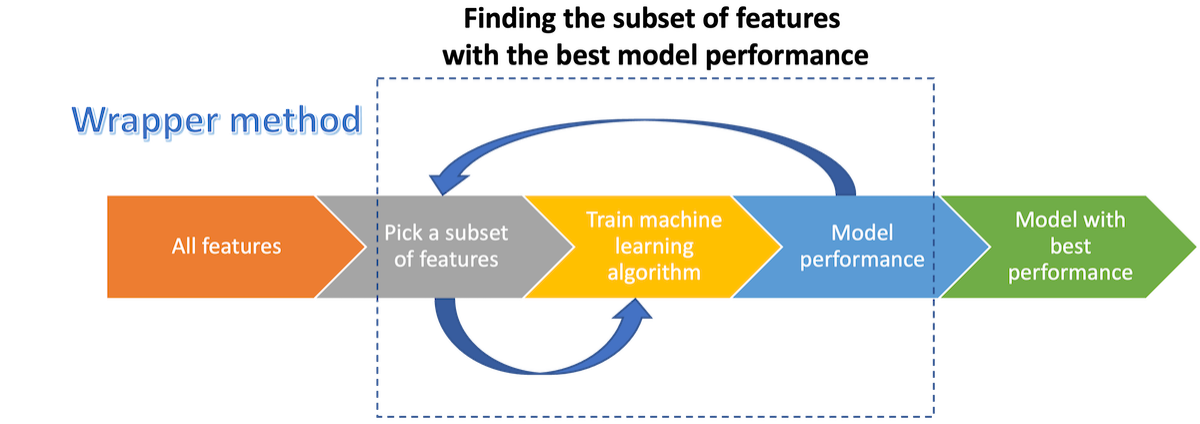
Steps of a wrapper feature selection method.

Wrapper methods try to use a subset of features while training a model. Changes will be made to the feature subset on the basis of the performance of the prior model (Figure 6). Therefore, finding the best features using the wrapper method is a search problem. These methods often have high computing costs [148]. Some of the most common wrapper methods are naive search, sequential forward feature selection, sequential backward feature selection, and generalized sequential search [149]. Some studies used this approach as their feature selection technique [72,75].

#### Filter Feature Selection Methods

**Figure 7:**
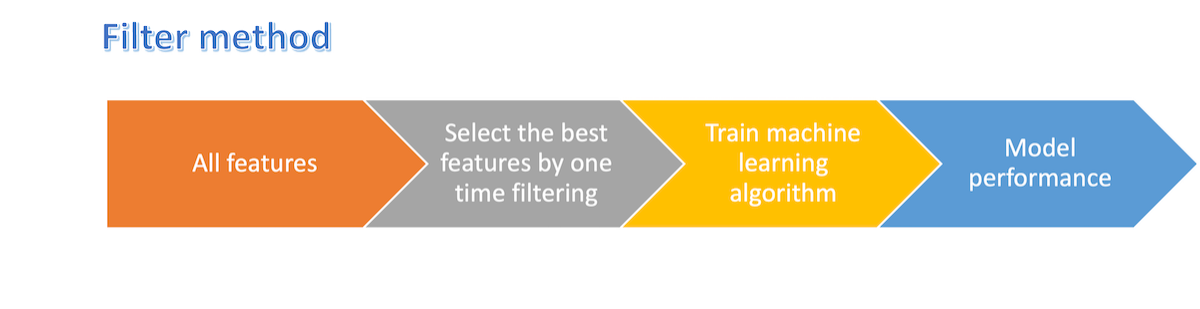
Steps of a filter feature selection method.

In general, filter methods are used as a preprocessing step without regard to any ML algorithms. Statistic tests are used instead to select features based on their correlation with dependent variables (Figure 7). The filter feature selection methods used in the literature are mentioned in subsequent sections.

#### Chi-Square Test

This test checks for independence between categorical features and the target variable. Features with high chi-square scores are selected, implying a strong association with the target variable, which may be valuable for the model (n=3, 3%) [56,135,150].

#### Pearson Correlation

Pearson linear correlation coefﬁcient (n=2, 2%**)** is a way to quantify how closely 2 sets of data are correlated linearly. It indicates how different measures are related to each other by a number between –1 and 1. Therefore, among highly correlated variables, some of them can be removed, as they do not add useful information to ML models [113,151].

#### Minimum Redundancy Maximum Relevance

**Figure 8:**
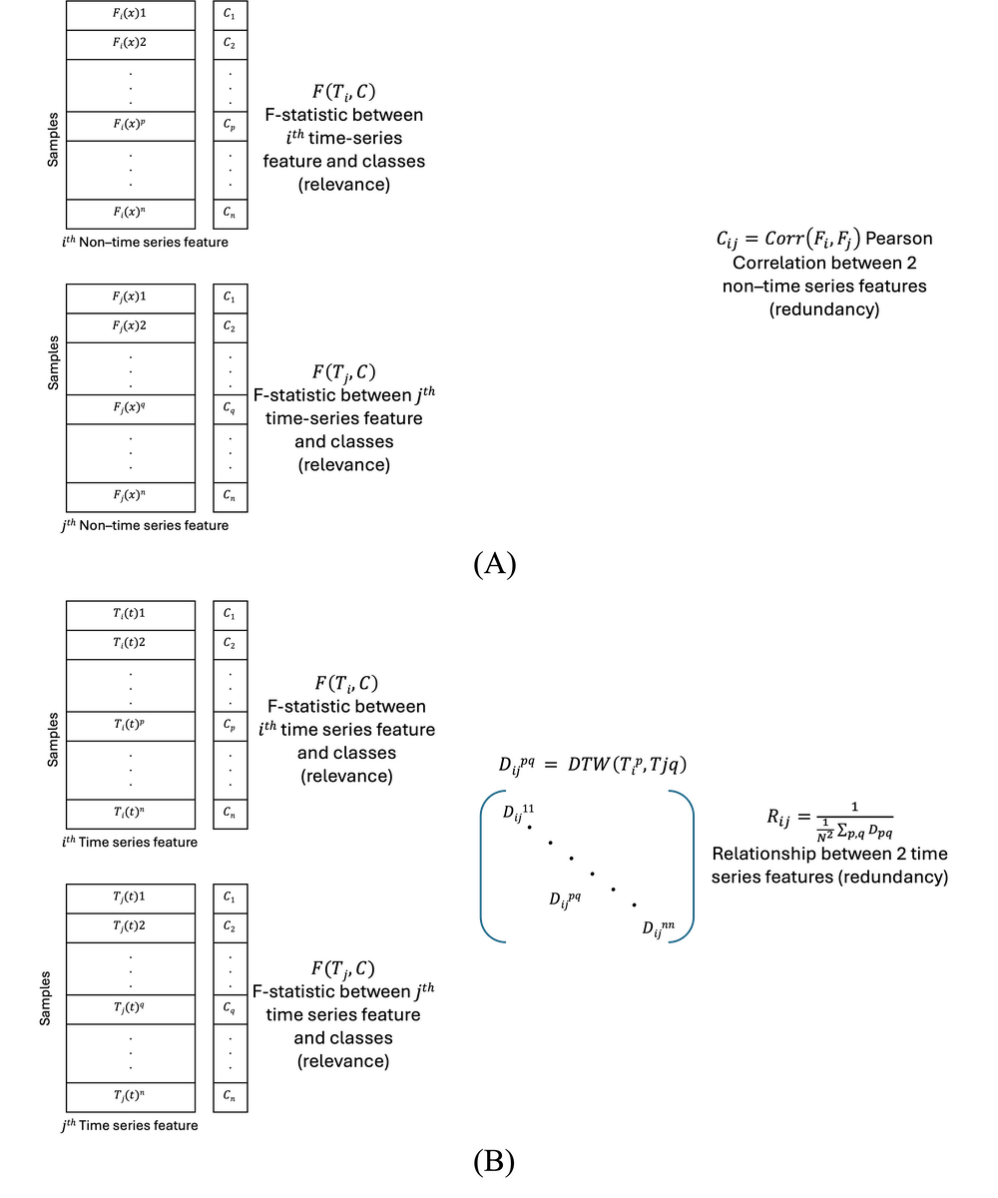
Calculation of relevance and redundancy for (A) nontime series features and (B) time-series features. DTW: dynamic time warping.

The maximum relevance minimum redundancy technique (n=2, 2%**)** chooses characteristics having a high correlation to output (relevance) and a low correlation to one another (redundancy). The *F* statistic is used to determine the correlation between features and the output, while the Pearson correlation coefficient (for non–time series features) and DTW (for time-series features) may be used to calculate the correlation between features (Figure 8). The objective function, which is a function of relevance and redundancy, is then maximized by selecting features one at a time using a greedy search. Mutual information difference and mutual information quotient criteria are both frequently used objective functions that depict the difference or quotient between relevance and redundancy [152,153]. Using this feature selection method, Giannakakis et al [130] have ranked ECG measurements in the order of importance as mean HR, LF, NN50, SD of HR, pNN50, LF or HF, RMSSD, HF, and total power.

### ML Techniques

The ML algorithms used for stress and MD detection have been reviewed in this section. The papers (n=98) used the DL approach or NN (n=38, 39%), LR (n=26, 27%), naive Bayes (NB; n=22, 22%), decision tree (DT; n=23, 23%), boosting (eg, adaptive boosting, extreme gradient boosting [XGBoost], etc; n=22, 22%), random forest (RF; n=37, 38%), discriminant analysis (DA; eg, linear DA and quadratic DA) (n=6, 6%), fuzzy C-means (FCM; n=2, 2%), k-nearest neighbors (KNNs; n=22, 22%), and support vector machines (SVMs; n=48, 49%). Table 2 shows the distribution of articles by ML model (refer to Table S1 in Multimedia Appendix 2 [29-31,33,36,39-43,56-63, 65,66,68,69,73,75-77,79,80,84,88,90,91, 94,99,100,105,107-112,114-117,119,122-129,137, 139,141,142,146,147,154-160] to find which papers have used each ML technique).

**Table 2:** Number of articles for each machine learning (ML) model (n=98).

ML model	Articles, n (%)
NN^a^	38 (39)
LR^b^	26 (26)
NB^c^	22 (22)
DT^d^	23 (23)
Boosting	22 (22)
AdaBoost^e^	8 (8)
XGBoost^f^	15 (15)
RF^g^	37 (38)
LDA^h^ and QDA^i^	6 (6)
Fuzzy	2 (2)
K-means	4 (4)
KNN^j^	22 (22)
SVM^k^	48 (49)
Other	19 (19)

^a^NN: neural network.

^b^LR: logistic regression.

^c^NB: naive Bayes.

^d^DT: decision tree.

^e^AdaBoost: adaptive boosting.

^f^XGBoost: extreme gradient boosting.

^g^RF: random forest.

^h^LDA: linear discriminant analysis.

^i^QDA: quadratic discriminant analysis.

^j^KNN: k-nearest neighbors.

^k^SVM: support vector machine.

#### LR Technique

LR (n=26, 27%) is a supervised parametric ML technique in which multiple independent variables will be used to detect the occurrence of stress or normal conditions [72,117]. Some studies used the numerical independent variables (eg, HRV time-domain features: RMSSD, HR, and pNN50) [94,155] or categorical data (eg, answers to multiple choice questions) obtained from questionnaires [107,114,115].

#### NB Algorithm

NB algorithm (n=22, 22%) is a supervised, generally parametric, classification method that uses the Bayes Theorem as its foundation and has the naive assumption of predictor independence. In other words, the NB classifier assumes that the existence of a given independent variable to predict the dependent variable is independent of the presence of any other independent variable that predicts the dependent variable.

#### DT Algorithm

**Figure 9:**
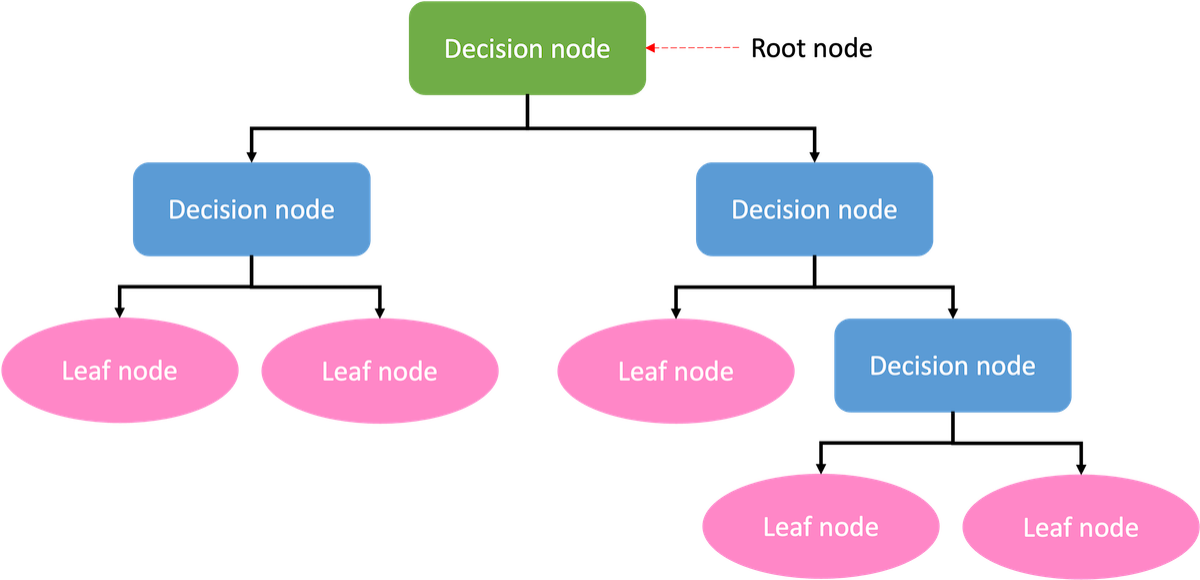
Structure of a decision tree.

The DT (n=23, 23%) is a supervised nonparametric ML algorithm used in classification and regression applications. It comprises a root node, branches, internal nodes, and leaf nodes in a hierarchical, tree-like structure (Figure 9).

#### Boosting Algorithm

Boosting (n=22, 22%) is an ensemble learning for reducing training errors by combining a group of weak learners. When using the Boosting algorithm, models are fitted on random samples of data, and then models are trained repeatedly in a sequence. When each model starts being trained in that sequence, it attempts to make up for the flaws of the one that came before it. The most commonly used Boosting algorithms are adaptive boosting, gradient boosting, and XGBoost.

#### RF Algorithm

RF (n=37, 38%) is a supervised nonparametric ensemble learning algorithm that uses many DTs built during the training process. The RF algorithm is used for both classification and regression problems. When it comes to classification, the RF’s output is the class that most of the DTs choose. For regression purposes, an individual tree’s predicted mean or average is returned as the output. Using RFs, we can overcome the tendency of DTs to overfit their training data.

#### DA Algorithm

DA (n=6, 6%) is a supervised parametric classification algorithm that works with data including a dependent variable and independent variables and is mostly used to classify the observation into a certain group based on the independent variables in the data. Linear DA and quadratic DA are the 2 forms of DA.

#### KNN Algorithm

**Figure 10:**
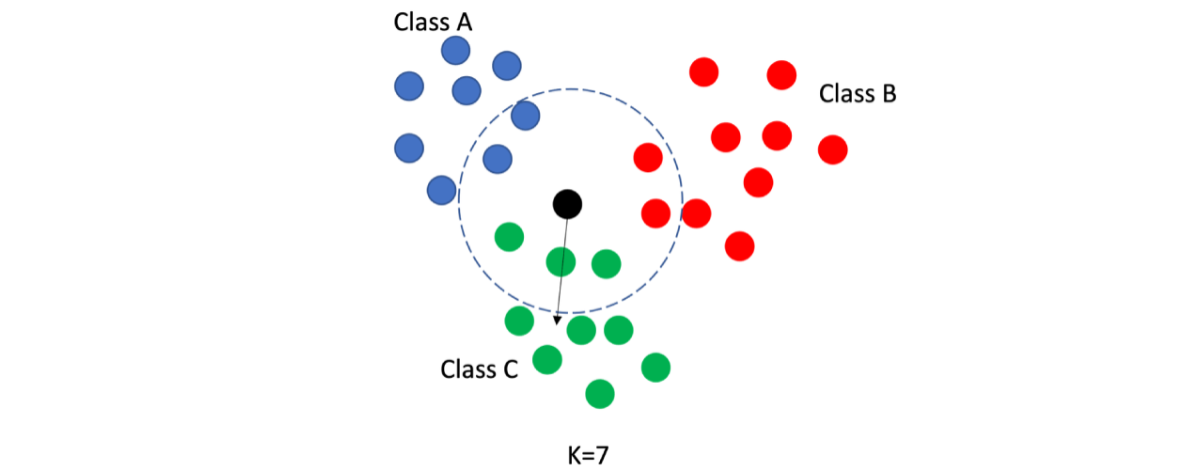
Example of k-nearest neighbor classification with K=7. In this example, the label of “Class C” is assigned to the new (black) datapoint since the majority of the 7-nearest datapoints to the new datapoint are from “Class C.”.

KNN (n=22, 22%) is a nonparametric supervised ML algorithm that is used for both classification and regression purposes. In classification, the algorithm determines the label of a new sample not available in the training data by assigning the label of the majority of k-nearest training data points to that new sample (Figure 10). In regression, the output for each sample is the average of the values of KNNs to that sample (not including the sample itself). In this literature, KNN has only been used for classification.

#### SVM Algorithm

**Figure 11:**
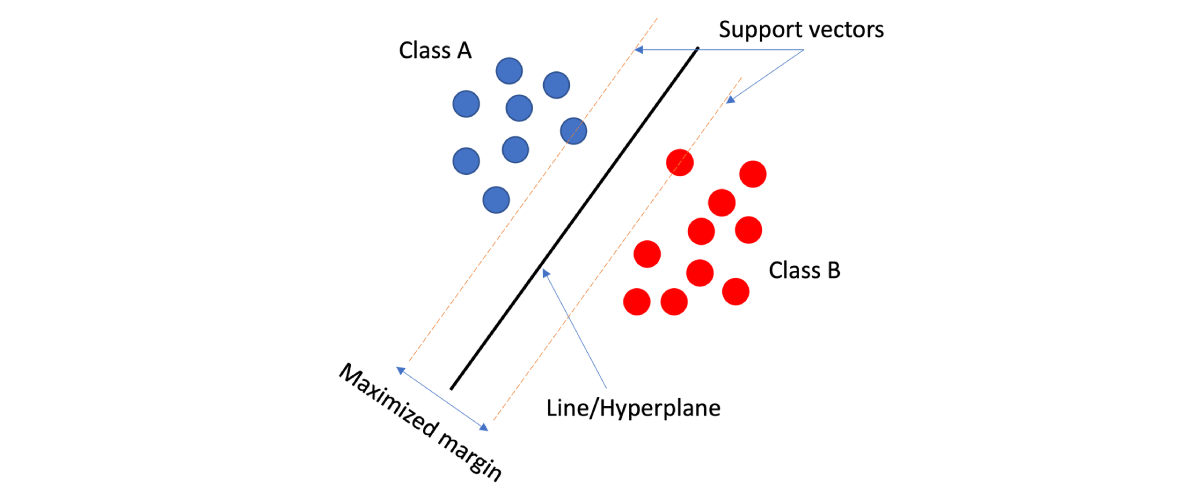
Visual representation of support vector machine algorithm.

SVM (n=48, 49%) is a parametric supervised ML algorithm used for both classification and regression problems. It can solve both linear and nonlinear problems using nonlinear kernels. For classification, the SVM algorithm finds a line (or a hyperplane for nonlinear kernels) between each pair of classes of the training data in a way that the margin distance of that line or hyperplane to the closest point of each of those 2 classes is maximized (Figure 11). This is repeated for all pairs of classes in the data set. Then, the obtained lines are used as boundaries for the classes. In regression, the SVM tries to find the line or hyperplane that within a very small margin of has the maximum number of data points. That line or hyperplane was used for regression.

#### K-Means Clustering

K-means clustering (n=4, 4%) is an unsupervised ML algorithm that aims to arrange objects into groups based on their similarity. To find those similarities, it calculates the distance of data points into K random cluster centroids and assigns each data point to its closest centroid. The location of each centroid is then updated by the average value of all data points associated with that centroid. This process is repeated until there is no change in the location of the centroids. In ML models for stress detection, K-means clustering has been used in the literature for the personalization of the ML models [58,67], and for labeling the data set [146,156].

#### NN Method

DL methods are a subset of ML methods, and NNs are at the heart of the DL algorithms. The NN (n=38, 39%) is a method for implementing ML that uses interconnected nodes or neurons arranged in a layered structure resembling the human brain. The different types of NNs have been explained in subsequent sections.

#### Artificial Neural Network

**Figure 12:**
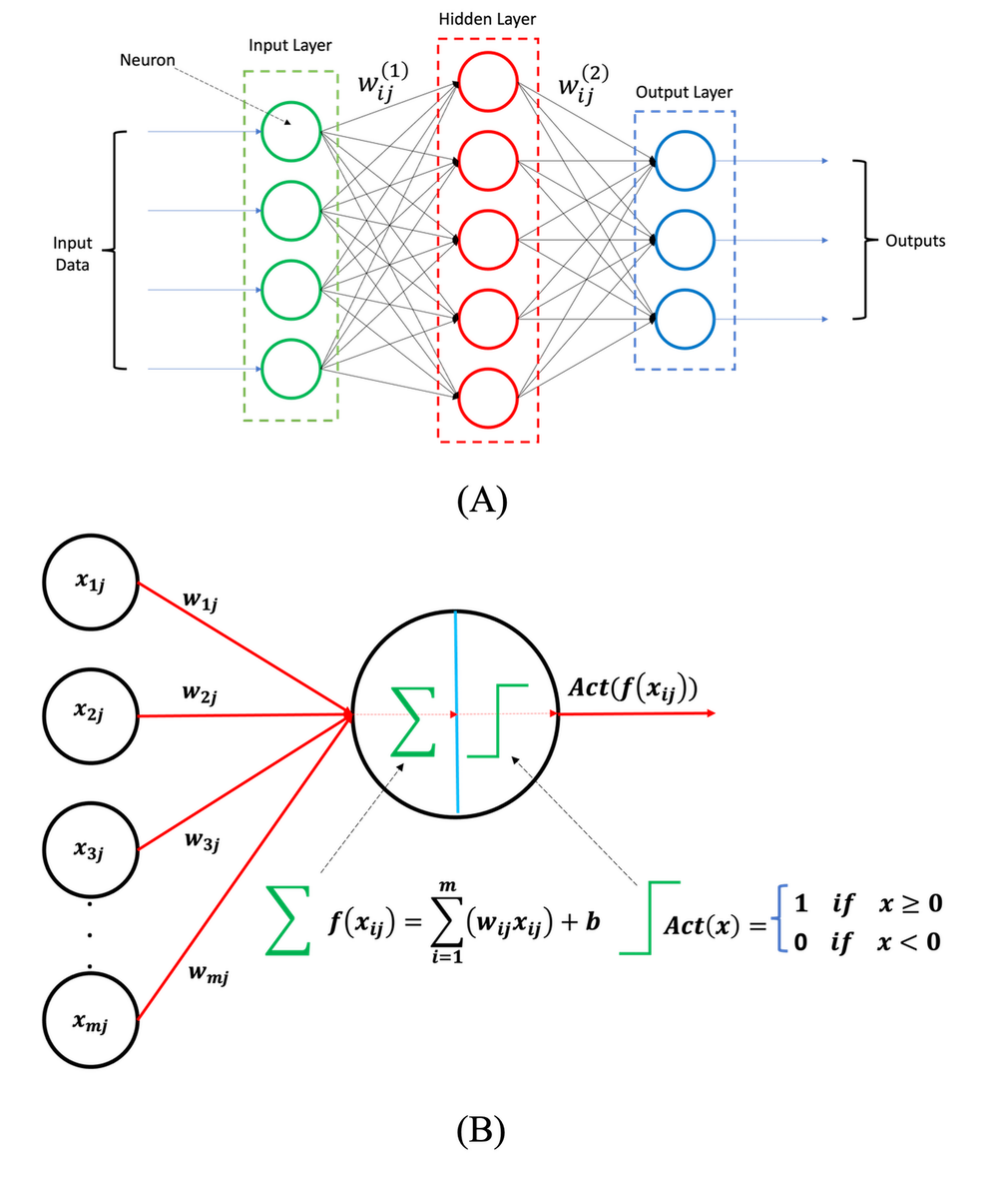
(A) Representation of an artificial neural network with 1 hidden layer. *W_ij_*^(1)^ and *W_ij_*^(2)^ denote the weights of the links connecting the first layer (input layer) to the hidden layer and the weights of the links connecting the second layer to the next layer (output layer), respectively. (B) Representation of how a single neuron works. First, all the outputs of the previous layer are multiplied by the weights associated with the links connecting them to the jth neuron of the next layer and summed by a bias (summation and bias step). The result is then passed through an activation function (activation step).

It is possible to think of a single perceptron (or neuron) as an abstract LR. In each layer of artificial neural networks (ANNs), a group of multiple perceptron or artificial neurons is used. Figure 12 shows an ANN with 1 layer and its working mechanism.

#### CNN Approach

**Figure 13:**
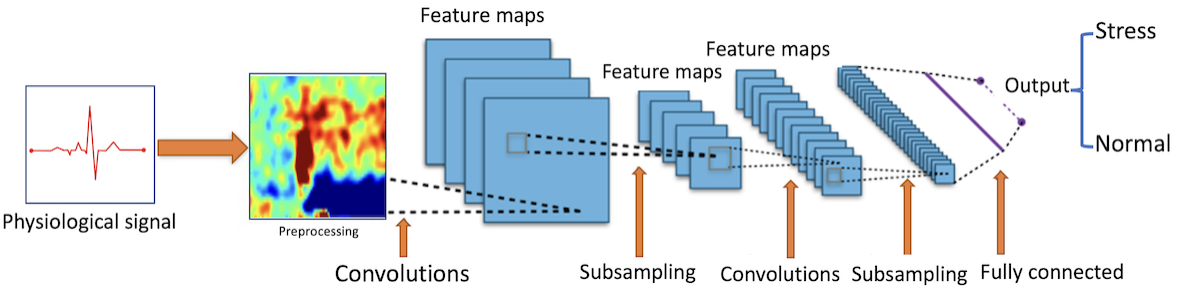
Representation of convolutional neural network for a physiological signal.

CNNs are a form of NN that is especially adept at handling data structures with a grid-like layout, such as images or objects. Classification and computer vision applications are common uses for CNNs (Figure 13).

#### Recurrent Neural Network Approach

A recurrent neural network (RNN) is a subset of ANNs designed specifically for use with time-series data and other sequence-based data. Long short-term memory (LSTM) networks are the most common type of RNNs. In RNNs, the attention mechanism is a method that simulates cognitive attention in NNs. The purpose of the impact is to encourage the network to give greater attention to the small but significant portions of the input data by enhancing some and reducing others. Since stress may alter a small portion of physiological data (eg, ECG), attention mechanisms can be used to detect stress using RNNs when large data sets are available [141].

Cong et al [102] introduced X-A-BiLSTM, which is a DL model that includes XGBoost (to filter data and handle imbalanced data) and attention Bi-LSTM (LSTM with forward and backward memory and attention mechanism) NN used for stress classification using text data.

#### Other ML Techniques

The total number of studies in this category include (n=19, 19%) of studies.

#### Voting Ensemble Classifier

The classification is decided based on weighted voting, which is determined by using a voting ensemble approach. The voting classifier allows for voting in which the final class labels are determined either by the class chosen most frequently by the classification models or by the average of the output probabilities from each classification model. In the literature, this method has been used for PTSD detection [127], stress, and stress-related MDs [84,95,118,156,157].

#### FCM Clustering

FCM is a clustering approach that assigns every data point to all the clusters with a certain probability instead of assigning each point to only 1 cluster. For instance, a data point that is near the cluster’s center will have a high degree of membership, while a data point that is distant from the cluster’s center will have a low degree of membership [158]. Since depression and anxiety are not discrete measures, some studies have used FCM as an alternative to other clustering techniques for the detection of these MDs [114,116].

## Discussion

### Principal Findings

In this review, the recent ML algorithms; preprocessing techniques; and data (eg, physiological data, questionnaire data, etc) used in the detection, prediction, and monitoring of stress and the most common MDs (ie, depression, anxiety, other stress-related MDs) have been reviewed.

On the basis of this review, it is concluded that among classic ML algorithms (excluding DL approaches), supervised models of SVMs and RF have been used more often and achieved better performance in terms of model accuracy and robustness (measured by parameters such as area under the receiver operating characteristic curve). The accuracy of ML models is a critical indicator of their utility in real-world applications. The review demonstrates that SVM consistently achieves high accuracy across various data types, including HR, HRV, and skin response. For instance, SVM achieved 93% accuracy with HR, PPG, and skin response data in the study by Nath et al [29] and 96% with skin response data in the study by Srividya et al [156]. These results underscore SVM’s robustness in handling complex, nonlinear data. RF also shows commendable performance, with an accuracy of 99.88% in the study by Trevisan [159], reflecting its strength in ensemble learning to mitigate overfitting and noise.

**Figure 14:**
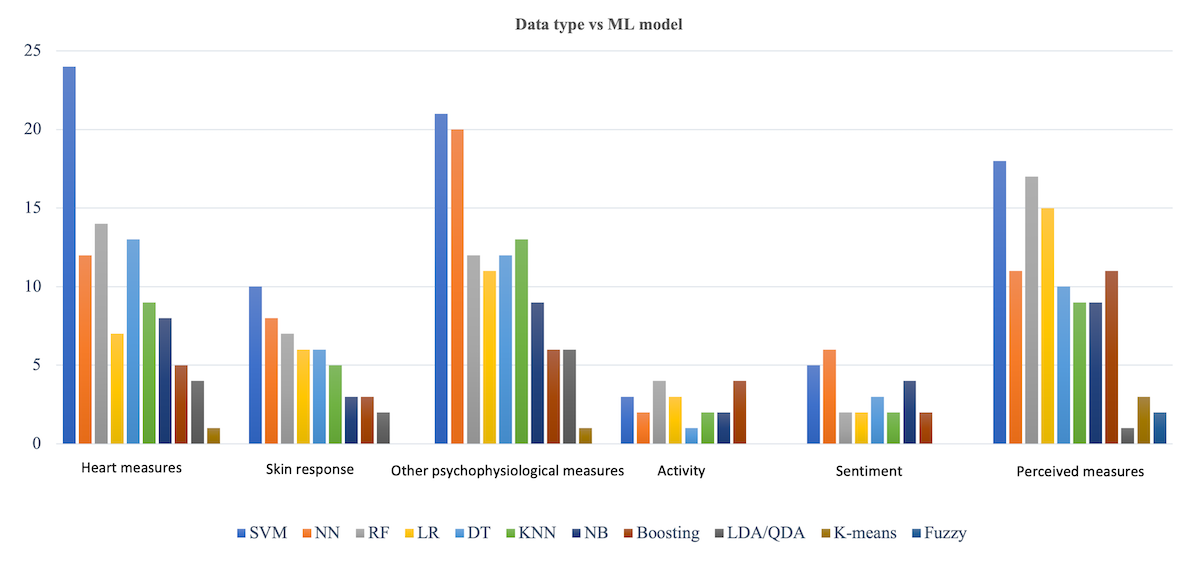
Distribution of machine learning (ML) models used for each type of data. In this figure, skin response and heart measures (including heart rate, heart rate variability, and blood pressure) have been shown separately because of their high use and importance in the literature. Other psychophysiological measures include electroencephalogram, electromyogram, eye tracking, and respiratory signals. Activity includes body movement. Sentiment data include speech and text data. Finally, perceived measures include questionnaires and self-report data. DT: decision tree; KNN: k-nearest neighbors; LDA: linear discriminant analysis; LR: logistic regression; NB: naive Bayes; NN: neural network; QDA: quadratic discriminant analysis; RF: random forest; SVM: support vector machines.

Moreover, among the predicting measures for stress and stress-related MDs, HR, HRV, and skin response have been used most often (Figure 14). These measures were the major explaining factors in the ML algorithms to predict stress and stress-related MDs. It is noticeable that DL approaches are becoming more popular as these techniques provide unique specifications that classic ML algorithms cannot provide.

Since stress is a time-dependent event, the relationship between different lags of time can be important for detection of stress. RNNs and CNNs will take into account the relationship between data points in different time series for their decision-making, and they have the potential to enhance the detections. DL models, specifically CNNs and LSTMs, show promising results, with CNNs achieving 92.8% accuracy in HRV and ECG data in the study by Quintero et al [155], indicating their potential in feature-rich physiological data. However, it is worth noting that DL models require substantial data for training, which may limit their applicability in studies with smaller data sets. Attention mechanism in RNNs is a new technique that is becoming popular for finding anomalies in physiological signals. However, based on the review of literature, this mechanism has only been used on text data (not on physiological signals) to detect stress. Therefore, the attention mechanism is the technology that can be further used for physiological signals to detect stress.

Unsupervised ML (and DL algorithms) such as clustering techniques have been used mostly for the preprocessing step to label the data (if labels are not available) and also for finding a representation of the data that achieves the best performance in detection algorithms.

For data preprocessing, feature selection (ie, filter and wrapper methods) and extraction techniques are commonly used. In feature extraction approaches, latent representations of data by transformations such as the output of encoder in autoencoders have been useful to remove data noises and to make the data more compact, making further computations more efficient. PCA and ICA are other most common feature extraction approaches used in the literature.

Among the selected features, statistical indicators of heart measurements such as the mean and SD of HR, along with time and frequency representations of HRV such as RMSSD and total LF and HF power, were most widely used. Heart measurements have also been used more often than other measurements, as they are unobtrusive, noninvasive, affordable, and easier to measure and describe a big portion of stress events. After those measurements, skin response measures have been found to be one of the most important factors in the detection of stress and its related disorders. The time-frequency approaches to analyzing time-series data are becoming more popular in this area as they are proper representations of data for DL approaches that can be more accurate and robust. As an example, for DL algorithms, RNNs with attention mechanisms can help to find portions of data related to stress and its related disorders with higher confidence.

Most of the study models do not interpret the ML models and look at them as black boxes. This limits the contribution to the body of science. Shapley additive explanations is a technique used by some studies to interpret the models such as the evaluation of features to find the most important ones and how in what direction each feature affects the predictions. Shapley additive explanations correlation plot provides insight into the distribution of the features themselves, as well as the relationship between their influence on the model. In other words, it provides the importance of each feature in the prediction of the dependent variable by considering both the main effect and the interaction effect of that feature with other features in the data [31,62,120,159,161,162].

Despite progress in stress detection methodologies, the exploration of personalized models has been limited. Most studies have not gone beyond basic normalization techniques, overlooking the fact that physiological measures are as distinct to individuals as biometric identifiers. A notable exception can be found in a select few studies [67,128,160], which have used more sophisticated personalization techniques, integrating complex data transformations to account for individual variability.

### Strengths of the Review

In undertaking this scoping review, we have embarked on a rich exploration of the applications of ML in the field of stress detection, articulating a narrative that is both comprehensive and detailed. The review lays out a landscape in which diverse data types are not merely cataloged but deeply analyzed for their roles and interconnections within the broader context of methodological approaches. This provides a robust understanding of the field’s current state and its complexities.

This review has documented a comprehensive assessment of various physiological measurement techniques, including HRV, EEG, ECG, and so on. This assessment is not just a recounting of the types of data used in the literature but a thoughtful consideration of how each contributes to a multifaceted understanding of stress indicators. It is an acknowledgment that the signals of stress are as complex as the condition itself, necessitating a rich palette of investigative tools.

The review also examines a range of advanced preprocessing techniques such as maximum relevance minimum redundancy, self-organizing map, synthetic minority oversampling technique, and PCA. This examination sheds light on how different studies leverage these methods to refine the quality of the data fed into ML models, thereby potentially enhancing the models’ accuracy and reliability in detecting stress. It is an illustration of how sophisticated data treatment can lead to more nuanced insights, even if our methodology did not directly use these techniques.

### Limitations

Our scoping review acknowledges its inherent constraints, including a possible selection bias due to potential omissions of pertinent studies. It serves as a contemporary cross-section of the rapidly evolving domains of ML and MH, underscoring the imperative for periodic scholarly review to sustain its relevance and precision. While we survey a broad spectrum of ML techniques applied to stress detection, we do not extensively assess their efficacy, suggesting a fertile ground for future empirical investigations to assess these methods across diverse data cohorts and settings. In addition, while we address the preprocessing techniques and their impact on model performance, our discussion does not delve into detailed technical analysis. Finally, the crucial issue of model interpretability is touched upon but not explored in depth, presenting an opportunity for further scholarly explorations.

### Conclusions and Future Directions

#### Overview

The pivotal insights from this review underscore the potential of ML to redefine the approach to MH care, particularly in the diagnosis and management of stress-related conditions and MDs. As we have discerned, there is an expansive field ripe for further exploration, with research gaps suggesting a number of promising directions. Guided by these insights, we can now chart a course for future research that not only expands the boundaries of our scientific understanding but also translates into tangible improvements in clinical practice.

#### Real-Time and Naturalistic ML Applications

The scarcity of real-time studies in naturalistic settings has highlighted the importance of developing ML models that accurately reflect and respond to the complexities of real life. Future research must prioritize the creation of algorithms capable of operating amidst the unpredictability of daily life, providing immediate insights and adaptable interventions. These models hold the potential to transform practice by offering tools that can preemptively identify stress and MD symptoms, enabling clinicians to intervene before conditions worsen.

#### Temporal Data and DL

Our review illuminates the untapped potential of time-series data in capturing the evolution of stress and MDs. DL techniques, specifically designed to interpret complex, sequential data, could lead to breakthroughs in how we understand and predict MH trajectories. For practice, this means more sophisticated diagnostic tools that can provide a nuanced picture of a patient’s MH over time, enabling personalized treatment plans that are responsive to the patient’s changing condition.

#### Personalization in ML Models

The need for individualized care for MH cannot be overstated. The heterogeneity of stress responses and MD symptoms calls for personalized ML models tailored to individual physiological and behavioral patterns. Future research should focus on leveraging multitask learning to refine algorithms that adapt to individual baselines, enhancing the personalization of care. For clinicians, this means access to tools that can more accurately reflect and respond to the unique needs of each patient, reducing the risk of misdiagnosis and improving treatment efficacy.

Predictive analytics can be instrumental in identifying key factors that contribute to misdiagnosis and delayed help seeking. Future studies should look to build on this knowledge to inform the creation of interventions that encourage timely and accurate diagnosis. In practice, this could lead to the development of targeted screening tools that assist clinicians in recognizing at-risk individuals more effectively. The integration of clinical expertise with ML innovation is crucial for the development of tools that are both advanced and clinically relevant. Collaboration between health care professionals, patients, and artificial intelligence developers will be essential in creating user-centered tools that address real-world needs. This collaborative approach will likely result in the development of artificial intelligence applications that are more intuitive and effective in clinical settings.

## Appendix

Multimedia Appendix 1 Search list for literature review.

Multimedia Appendix 2 Comparison between machine learning models.

Multimedia Appendix 3 PRISMA-ScR Checklist.

## Abbreviations

ANN: artificial neural network
ASR: artifact subspace reconstruction
BP: blood pressure
CNN: convolutional neural network
DA: discriminant analysis
DL: deep learning
DT: decision tree
DTW: dynamic time warping
ECG: electrocardiogram
EEG: electroencephalogram
EMG: electromyogram
FCM: fuzzy C-means
HF: high frequency
HR: heart rate
HRV: heart rate variability
ICA: independent component analysis
KNN: k-nearest neighbor
LF: low frequency
LR: logistic regression
LSTM: long short-term memory
MD: mental health disorder
ML: machine learning
NB: naive Bayes
NN: neural network
PCA: principal component analysis
PPG: photoplethysmogram
PRISMA-ScR: Preferred Reporting Items for Systematic Reviews and Meta-Analyses extension for Scoping Reviews
PSD: power spectral density
PTSD: posttraumatic stress disorder
RF: random forest
RMSSD: mean square of successive RR interval differences
RNN: recurrent neural network
SVM: support vector machine
XGBoost: extreme gradient boosting
